# Developing a novel optimisation approach for keeping heterogeneous diets healthy and within planetary boundaries for climate change

**DOI:** 10.1038/s41430-023-01368-7

**Published:** 2023-11-21

**Authors:** Patricia Eustachio Colombo, Liselotte Schäfer Elinder, Esa-Pekka A. Nykänen, Emma Patterson, Anna Karin Lindroos, Alexandr Parlesak

**Affiliations:** 1 Department of Global Public Health, Karolinska Institutet, Stockholm, Sweden; 2 Centre on Climate Change and Planetary Health, London School of Hygiene and Tropical Medicine, WC1E 7HT London, UK; 3 Centre for Epidemiology and Community Medicine, Region Stockholm, Stockholm, Sweden; 4 Functional Foods Forum, University of Turku, Turku, Finland; 5 The Swedish Food Agency, Uppsala, Sweden; 6 Department of Internal Medicine and Clinical Nutrition, the Sahlgrenska Academy, University of Gothenburg, Gothenburg, Sweden; 7 Department of Nutrition, Exercise and Sports, Copenhagen University, Copenhagen, Denmark; 8 Personalized Nutrition, Duale Hochschule Baden-Württemberg, Heilbronn, Germany

**Keywords:** Risk factors, Malnutrition

## Abstract

**Background and objectives:**

Current dietary habits have substantial negative impacts on the health of people and the planet. This study aimed to develop a novel approach for achieving health-promoting and climate-friendly dietary recommendations for a broad range of consumers.

**Subjects and methods:**

Hierarchical clustering analysis was combined with linear programming to design nutritionally adequate, health-promoting, climate-friendly and culturally acceptable diets using Swedish national dietary data (*n* = 1797). Diets were optimised for the average consumption of the total population as well as for the dietary clusters.

**Results:**

Three dietary clusters were identified. All optimised diets had lower shares of animal-source foods and contained higher amounts of plant-based foods. These dietary shifts reduced climate impacts by up to 53% while leaving much of the diet unchanged. The optimised diets of the three clusters differed from the optimised diet of the total population. All optimised diets differed considerably from the food-group pattern of the EAT-Lancet diet.

**Conclusions:**

The novel cluster-based optimisation approach was able to generate alternatives that may be more acceptable and realistic for a sustainable diet across different groups in the population.

## Introduction

Contemporary diets in high and middle income countries are major contributors to the burden of chronic diseases as well as to the rapidly accelerating climate crisis [1]. The global food system–from production to consumption—thus needs a revamp to meet the 2015 Paris Agreement on climate change [2] and the Sustainable Development Goals. In a market economy, demand and supply of food are closely connected, making consumers’ eating behaviours one of the most important factors contributing to human and environmental health [3].

The EAT-Lancet Commission has suggested a healthy reference diet that would also help keep the global food system within six environmental planetary boundaries [1]. It emphasises a ‘plant-forward’ diet dominated by whole grains, fruits, vegetables, nuts and legumes where meat and dairy constitute a small or negligible part. Despite this robust evidence, there is currently no consensus on *how* to operationalise these dietary targets and achieve acceptability among consumers in different population groups with diverse cultural backgrounds [4].

For most high-income populations, adoption of the EAT-Lancet diet would imply a significantly higher share of plant-based foods while markedly reducing the intake of animal-based products [5]. To account for both nutritional and environmental demands as well as affordability, holistic approaches such as optimisation analysis with linear programming (LP) have been used for a wide range of settings [6, 7]. To also consider the cultural acceptability of optimised diets, the deviation from the reported average diet of the total population has been minimised [6, 8–11]. However, delivering one “acceptable” solution based on the average consumption of different foods or food groups may imply minor dietary changes for some individuals but larger and potentially unrealistic changes for several groups in the population [12–14]. For example, male individuals in European countries are likely to face larger absolute and relative changes to their consumption of red/processed meat as compared to females given their different needs and baseline consumption levels [15]. Hence, developing any type of food-based advice or guidance by optimisation of the average diet is likely to overlook the heterogeneity of diets within populations [16]. There is thus a need to explore if altering current optimisation approaches could lead to solutions that better reflect the dietary variability in a given population.

The primary aim of this study was to optimise the diet of groups in the population with different eating patterns and to see if this provides a more realistic approach than optimising for the national average consumption. Diets were optimised to meet nutritional requirements, food-based dietary guidelines (FBDGs) and a limit for food related greenhouse gas emissions (GHGE) of 1.57 kg/day as suggested by the Intergovernmental Panel on Climate Change (IPCC) [17]. We also compared the optimised diets to the proposed EAT-Lancet diet [1].

## Materials and methods

### Study design and dietary data

This was a modelling study combining hierarchical clustering analysis with linear programming to design nutritionally adequate, health-promoting, climate-friendly and culturally acceptable diets. Self-selected diets were derived from the nationally representative Swedish dietary survey Riksmaten Vuxna 2010–11 (Riksmaten Adults) [18]. The data, which were collected between May 2010 and May 2011 by the Swedish Food Agency, is publicly available in fully anonymised form [19]. Briefly, a web-based 4-day diary was completed by 1797 adults aged 18–80, and all foods and drinks consumed over four consecutive days were recorded. The participants were able to choose from more than 1900 different food items and dishes and several portion sizes. The study sample consisted of 56% females and the mean age was 48 years. Information on income and other sociodemographic factors was also gathered. A more detailed description of the material and methods used for this study can be found in the Supplementary Information.

### Nutritional composition

Energy and nutrient intakes of the edible parts of foods as eaten (e.g., cooked pasta) were automatically calculated through linkage with the Swedish Food Agency’s Food composition database version Riksmaten Vuxna 2010–11.

### Climate footprints

The carbon dioxide equivalents (CO_2_eq) of foods were derived from the Climate Database developed and maintained by the Research Institutes of Sweden (RISE) [20], which is linked to the Swedish Food Agency’s Food composition database. The database includes CO_2_eq estimations for 2078 food items following life-cycle assessment standards [21, 22] taking into consideration Swedish production and consumption patterns [20]. The CO_2_eq estimations consider the impact from carbon dioxide (CO_2_); methane (CH_4_); and nitrous oxide (N_2_O), which have been weighted in line with their respective global-warming potential over a 100 year period using factors recommended by the IPCC [23]. The CO_2_eq data did not take into consideration the packaging, transportation from stores to households, meal preparation or food waste.

### Cost of foods

The webpage “Matpriskollen” [24], which compares the prices of foods among twelve of Sweden’s largest food retailers, was used to estimate the price of each food in the year 2020. An average price was calculated for each food item based on varying available prices for a food item (including low price, conventional and organic varieties).

### Grouping of foods

For analytical and descriptive purposes, foods were grouped in 24 food categories, based on the categorisations used in the RISE Climate Database: Red meat (including red meat dishes); Processed meat (both red meat and poultry); Poultry (including poultry based dishes); Seafood (including fish, mussels and crabs, and seafood dishes); Offal; Dairy (e.g., milk and cheese); Eggs; Pasta and rice dishes with meat/fish (e.g., composite dishes like lasagne); Pasta and rice dishes with dairy/eggs (e.g., composite dishes like vegetarian lasagne); Vegetable oils; Vegetables (whole vegetables and a few vegetable based dishes); Potatoes (including potato based dishes); Pulses (beans, lentils, peas and chickpeas); Fruits and berries (including smoothies); Nuts and seeds; Meat alternatives (e.g., soy mince); Dairy alternatives (e.g., oat milk); Mixed/animal fats (added fats such as butter, margarine-butter mix); Cereals/grains (including e.g., breakfast cereals and, pasta); Rice; Savoury snacks; Sugar and sweets (including chocolate); Drinks other than milk; and Other (e.g., seasonings and sauces). Further details on the categorisation can be found elsewhere [20].

The foods in the baseline and optimised diets were additionally re-grouped in order to be comparable to the EAT-Lancet Commission’s food categorisation [1], namely: Whole grains (rice, wheat, corn and other); Tubers or starchy vegetables (including potatoes); Vegetables; Fruits; Dairy foods (whole milk or equivalents, including butter); Beef, lamb and pork; Chicken and other poultry; Eggs; Fish; Legumes; Nuts; Added fats (unsaturated oils and saturated oils); and Added sugars. This categorisation was either based on the most dominant component or calculated based on the proportional shares, based on recipes.

### Cluster analysis

Clusters analysis was performed to identify dominating eating patterns in the Swedish population. Firstly, the R package clValid [25] was applied to the dietary data to simultaneously compare multiple clustering algorithms and clustering methods. By comparing the discriminatory power of different calculation paths, clValid identified hierarchical clustering to be the best fitting clustering algorithm for our data. It also proposed using Canberra distances with Ward’s method in a hierarchical clustering as this combination resulted in the highest value for Dunn’s Index (the ratio of the smallest distance between observations not in the same cluster to the largest intra-cluster distance). Secondly, the NbClust package in R [26] (which uses 30 different indices to suggest the best clustering approach and number of clusters to choose based on all combinations of self-organising clusters, distance measures, and clustering methods) was used to determine the optimal number of clusters when combining Canberra distances with Ward’s method (results suggesting 2 or 3 clusters, visualised in Supplementary Fig. 1). Following on these initial exploratory analyses, data was scaled and hierarchical clustering using Ward’s method and Canberra distances was applied to the dietary data. Based on the outputs from NbClust, three clusters were chosen for this analysis.

Food groups that were consumed by less than 75% of the population were not included in the clustering to avoid bias emerging from missing data. Two exceptions were made for the food groups Pulses and Nuts and Seeds, since these food groups are seen as indicators of both climate friendliness and healthy eating [1]. Hence, the following food groups were included in the clustering: Red meat, Processed meat, Vegetables, Fruits and berries, Dairy, Pulses, Nuts and seeds, Seafood, Mixed animal fats, Sugar and sweets, Rice, Potatoes, Cereals/grains, Eggs, and Poultry. Whole grains were also included in the clustering although not classified as a food group in the food consumption survey. For the clustering procedure, intakes of food groups were standardised for individual energy intake (g/MJ) to account for heterogeneous energy intake.

### Comparing the clusters

Clusters were compared post-hoc on the basis of the energy-adjusted intake of the food groups included in the cluster analysis (g/MJ), age (y), income (SEK), sex (male/female), and CO_2_eq (g/MJ). Kruskal–Wallis test was used to statistically determine if significant differences between clusters existed with regards to food groups, CO_2_eq and income since these variables were not normally distributed. Age was normally distributed and thus assessed with Analysis of Variance. Sex (categorical variable) was assessed using Pearson’s chi-squared test. As for the non-normally distributed variables, the Dunn (1964) Kruskal–Wallis test for multiple comparison (alpha adjusted with the Benjamini-Hochberg correction) was used as a post-hoc test to identify which clusters that differed significantly. Tukey’s honest significance test was applied as a post-hoc test for the normally distributed variables. Statistical significance was set at *P* ≤ 0.05. Both the cluster analysis and all statistical computations were performed in R version 4.1.1 [27].

The healthiness of the three clusters was calculated in accordance with a previously developed healthy eating index relevant for the Swedish context – SHEIA15 [28]. The ratio between the baseline intake and the recommended intake of nine different dietary components were accordingly calculated (Supplementary Table 1) and summed to a total score. Ratios <0 and >1 were recoded to zero and one, respectively, resulting in a range of 0–9. As previously suggested [28], the summed ratios for the different dietary components were categorised into three defined levels; low (<4 points), medium (4–7 points), and high (>7 points).

### Optimisation

The chosen optimisation method of LP has successfully been applied to optimise goal determinants of diets while considering a multitude of (sometimes conflicting) constraints [6, 29]. Briefly, it is the application of an algorithm for either maximising or minimising a specific linear objective function (the variable being optimised) which is subjected to a set of linear constraints (predetermined requirements that should be met) on a list of decision variables (in this case, the absolute amount of each individual food item) [30]. A feasible solution is found when all constraints are met. If the selected constraints are too rigorous, the algorithm will not be able to provide a solution, i.e., there will be no feasible solution to the mathematical problem. The constraints that determine the objective function’s capacity to be minimised or maximised (i.e. those conditions fulfilled by 100% in relation to its predetermined limit) are considered “active constraints” [31]. Linear optimisation was performed with the CBC (COIN-OR Branch and Cut) Solver algorithm, which is part of the Excel® 2016 software add-in OpenSolver, V. 2.9.0 [32].

We optimised the average diet of the total study sample (*n* = 1797, i.e. the “TotPop” diet) as well as the diet of the three clusters (Table 1), respectively. The relative deviation (RD) from the reported intake of each food item was calculated as RD (*w*_opt_ – *w*_rep_)/*w*_rep_, where w_opt_ is the food weight in the optimised diet and *w*_rep_ is the reported intake. As the objective function of all LP models, we chose the minimisation of the total relative deviation (TRD) from the baseline diet [10, 11]. This objective function was implemented to maximise the similarity between the baseline and the optimised diet solutions. The decision variables were the amounts of individual food items in the total study sample/each cluster. All optimisations applied dietary reference values (DRVs), covering the nutritional needs of 97.5% of the population and based on the Nordic Nutrition Recommendations 2012 [33], as obligatory constraints (Supplementary Table 2). In cases where the DRVs differed depending on sex, the nutritional constraints were weighted according to the DRVs and population size of the sex groups in the study sample. Total daily energy (kcal) was set to equal the baseline energy intake within the total population/the three clusters in all models (Supplementary Table 2). All models were also constrained to meet the Swedish Food Based Dietary Guidelines (FBDGs) (Table 1) [34]. Individual food items were allowed to be reduced to 0 g; however, they were not allowed to increase by more than 200% relative to their respective baseline weight. This constraint was applied to all foods except for the ones belonging to the food groups Pulses, Nuts and seeds, Dairy substitutes, Meat substitutes and Vegetable oils. Because of their plausible role in making up a healthy and environmentally friendly diet and their partly recent appearance on the market, these foods/food groups were allowed to increase by any value.

**Table 1:** Characteristics of all applied models.

	Model names	Objective function (minimum)	Decision variables	Swedish Food Based Dietary Guidelines	Nutritional constraints	Acceptability constraint	CO_2_eq constraint
1st set of models (nutrient restricted)	TotPop Classic NutRich LowClim	TRD^a^ from baseline diet	Amount of individual foods	≥500 g fruit and vegetables/day ≥45 g seafood/day ≥75 g/10MJ of whole grains/day ≤71 g red/processed meat/day	Meet all DRVs^b^	Max RD^c^ for food items set to +200%^d^	na
2nd set of models (nutrient + CO_2_eq-restricted)	TotPop+ Classic+ NutRich+ LowClim+						Max. 1.57 kg CO_2_eq

*na* not applied.

^a^Total Relative Deviation.

^b^Dietary Reference values, i.e., estimated energy requirements (EERs), recommended intake ranges for macronutrients, recommended intakes (RIs) for micronutrients [33].

^c^Relative Deviation from baseline food consumption.

^d^Food groups Pulses, Nuts and seeds, Dairy substitutes, Meat substitutes and Vegetable oils exempt from this limitation.

In a first set of models, all aforementioned constraints, but no upper threshold for the associated GHGE, were applied. The second set of models also included a limit for total diet-related CO_2_eq. These models were constrained to contain less than or equal to 1570 g of CO_2_eq per day. The cost of the baseline and optimised diets was calculated separately and was not included as a constraint in the models. The average relative deviation (ARD) from the baseline food consumption (i.e., the TRD divided by the total number of food items included in the model) was calculated as an output and used as a proxy of similarity between the baseline and the optimised food consumption and as an assumed indicator of cultural acceptability. Active nutrient constraints (those meeting exactly 100% of the applied limit [31]) were identified for each solution. A more detailed description of the optimisation procedure can be found in the Supplementary Information.

## Results

### Identifying prevalent dietary clusters

The cluster analysis resulted in three diet clusters roughly balanced in size (707, 534 and 556 individuals in clusters 1, 2 and 3 respectively). Supplementary Fig. 2 displays the hierarchical relationships between study participants. The three clusters differed significantly in their median daily consumption (g/MJ) of all food groups part of the cluster analysis, median daily dietary CO_2_eq (g/MJ), median yearly income, mean age, and sex distribution (Supplementary Tables 3 and 4). Based on these observed differences, the following classification of the clusters was made:

- Cluster 1 – “the Classic Baseline diet”: High inclusion of foods of a typical Swedish diet (red and processed meat, and potatoes), low inclusion of fruits and vegetables, high CO_2_eq emission, medium SHEIA15 (Swedish healthy eating index)
- Cluster 2 – “the NutRich Baseline diet”: High inclusion of nutrient dense animal products, nuts and vegetables, highest CO_2_eq emission, high SHEIA15
- Cluster 3 – “the LowClim Baseline diet”: High inclusion of low GHGE-foods with favourable nutritional properties (vegetables, pulses) and, to some extent, less favourable (sugar and sweets), lowest CO_2_eq emission, high SHEIA15

### Baseline diets

The CO_2_eq emissions of the baseline diets ranged between 2770 (LowClim Baseline) and 3361 (Classic Baseline) g/day (Table 2). All baseline diets contained lower than recommended amounts of carbohydrates, dietary fibre, and iron (Supplementary Table 5). They were also lower than recommended with respect to the DRV for vitamin D, except for the LowClim Baseline diet which met this DRV by 100%. All baseline diets exceeded the recommended amounts of saturated fatty acids and sodium (Supplementary Table 5). The cost of the four baseline diets ranged between SEK 65 and 68 (approximately 6.5 USD/person/day) (Table 1).

**Table 2:** Crude CO_2_eq values, cost, average relative deviation (ARD), and the number of foods removed, reduced or increased in the optimised diets of the total study sample (*n* = 1797) as well of the three clusters, respectively, compared with their baseline diets.

Diets^a^	CO_2_eq	Change in CO_2_eq	Cost	ARD	Foods available	Foods removed	Foods reduced	Foods increased
	g	%	SEK^b^	%	#	#	#	#
TotPop baseline	3104	na	67	na	1665	na	na	na
TotPop	2771	−11	71	3.6	1665	34	1	15
TotPop+	1571	−49	61	6.7	1665	79	3	17
Classic baseline	3361	na	68	na	1399	na	na	na
Classic	2568	−24	67	19.7	1399	65	4	58
Classic+	1571	−53	59	22.8	1399	102	5	61
NutRich baseline	3110	na	65	na	1404	na	na	na
NutRich	2780	−11	67	3.0	1404	25	2	10
NutRich+	1571	−49	59	6.7	1404	67	3	13
LowClim baseline	2770	na	67	na	1416	na	na	na
LowClim	2536	−8	69	2.6	1416	21	3	8
LowClim+	1571	−43	60	5.8	1416	60	3	12

*ARD* average relative deviation within each model, which indicates the average change per food item from the reported dietary intake, *SEK* Swedish Krona, *na* not applicable.

^a^Model acronyms without a “+” have Dietary Reference Values and the Swedish Food Based Dietary Guidelines as obligatory constraints. Model acronyms with a “+” additionally include a CO_2_eq limit of 1.57 kg CO_2_eq/day as an obligatory constraint.

^b^SEK = Swedish Krona, (1 SEK equals to~0.1 USD).

### Optimised diets

In the optimised isocaloric diets meeting DRVs and the Swedish FBDGs only (TotPop, Classic, NutRich and LowClim models), GHGE were reduced by 8–24% compared with the baseline diets (Table 2). The cost increased slightly (~1–3%), and average relative deviations (ARDs) were low (~4%) for most of these diets. The exception was the Classic diet, which had a marginally lower (−1%) cost and an ARD of about 20%. The number of foods removed, reduced or increased was fairly similar across the optimised diets. However, more foods in the Classic diet were changed compared to the other ones.

Adding the upper CO_2_ constraint of 1.57 kg CO_2_eq/person/day [17] (TotPop+, Classic+, NutRich+ and LowClim+ models) reduced diet-related GHGE by 43–53% (Table 2). Compared to baseline, the diet cost was reduced approximately by 8–13% in all these optimised diets (Table 2). The inclusion of the CO_2_eq constraint increased the ARDs only slightly for all diets, ranging from 5.8 % in the LowClim+ diet to 22.8% in the Classic+ diet.

**Fig. 1:**
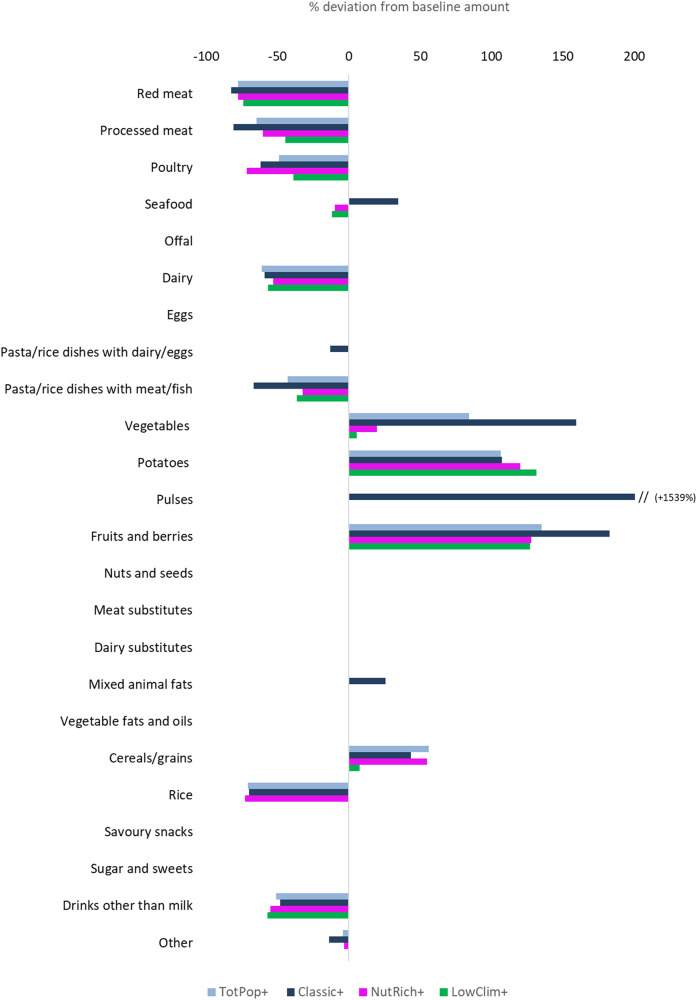
Relative (%) deviation from baseline intakes of different food groups according to the optimised dietary models. The presented optimised dietary models include constraints on dietary reference values, Food Based Dietary Guidelines and CO_2_eq. The coloured bars represent the % deviation from baseline intakes (0 on the X axis) for the optimised average diet (TotPop+) and the three clusters (Classic+, NutRich+, and LowClim+). For the Classic+ diet, the relative deviation was +1500%.

All optimised diets constrained to meet nutritional, FBDG and CO_2_eq targets had lower shares of animal-based foods (Fig. 1). The Classic+ diet contained 82% less Red meat, 81% less Processed meat, 62% less Poultry, and only about one third of the Dairy compared to its baseline amounts (Fig. 1). The TotPop+, NutRich+ and LowClim+ diets also contained considerably less Red/Processed meat. In contrast to the Classic+ diet, the other optimised diets did not show increases in Seafood (Fig. 1). The optimised diets contained higher amounts of Vegetables (+6 to +159%), Potatoes (+106 to +131%), and Fruits and berries (+127 to +183%). The greatest changes in Cereals/grains occurred in the Total+ diet (+56%) whereas the LowClim+ diet experienced only a moderate change ( + 8%) (Fig. 1). Rice was reduced by ~70% in all optimised diets except for the LowClim+ diet, where this food group remained unchanged. A noticeable (15-fold) increase in Pulses was observed in the Classic+ diet only. A more detailed presentation of each food group associated with the baseline and/or optimised clusters is found in Supplementary Tables 6–10. Iron and vitamin D were active lower-threshold constraints while added sugars and sodium were active upper-threshold active constraints in almost all models (Supplementary Table 5).

### Optimisation of total diet vs. clustering approach

**Fig. 2:**
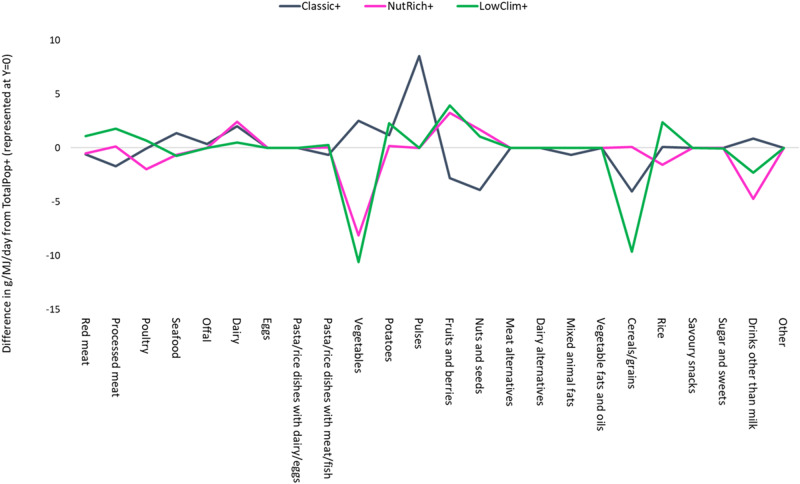
Difference between the absolute change (baseline vs. optimised) in daily energy-adjusted intake (g/MJ) of different food groups in each cluster and the absolute change (baseline vs. optimised) in daily energy-adjusted intake (g/MJ) of these food groups in the TotPop+ model. Here, Y = 0 represents the TotPop+ diet and the horizontal lines represent how much each cluster-specific diet deviates in terms of the dietary changes required to meet all nutrient-, Food Based Dietary Guideline-, and CO_2_eq constraints.

Figure 2 was developed to explore whether a diet optimised based on the average diet of the entire sample would result in a dietary pattern equal to the diets of the optimised clusters. Figure 2 illustrates how much each of the optimised cluster diets (Classic+, NutRich+, and LowClim+) differ from the diet optimised based on the average intake of the total population (TotPop +). Values indicate the absolute difference between the baseline vs. optimised energy-adjusted intake (g/MJ/day) of different food groups—i.e., the dietary change resulting from optimisation—in the TotPop+ model compared against the dietary change resulting from optimisation in each cluster. For example, the TotPop+ model requires an increase in cereal consumption of 10.5 g/MJ/day whereas individuals belonging to the Classic cluster need to increase their Cereal intake by only 7.5 g/MJ/day. Hence, the resulting difference (−3 g/MJ/day) is shown in the graph. Overall, the three cluster-specific diets face dietary shifts that differ from those demanded by the TotPop+ model.

### Optimised diets vs. the EAT-Lancet diet

**Fig. 3:**
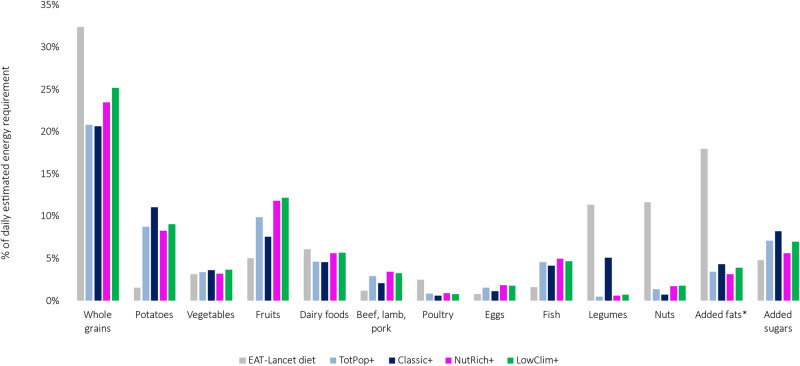
Comparison between the EAT-Lancet diet and the optimised diets of Swedish Adults. Columns represent the percent of daily estimated energy requirement for different food groups in the EAT-Lancet diet and in the four fully optimised diets (TotPop+, Classic+, NutRich+, LowClim+). Food categories used in this comparison were based on the ones used for the EAT-Lancet diet [1]; *Added fats exclude dairy-based fats (such as butter), which are included in “Dairy foods”.

Overall, the EAT-Lancet diet was higher in Whole grain foods, Dairy, Poultry, Legumes, Nuts, and Added fats, but lower in Potatoes, Fruits, Red/processed meat, Eggs, Fish and Added sugars than that provided by the optimised diets and expressed as a percentage of total energy intake (Fig. 3). However, all optimised diets matched the EAT-Lancet diet with regards to Vegetables. The NutRich+ diet was close to matching the EAT-Lancet diet in terms of Added Sugars whereas the LowClim+ diet was closest with respect to Whole grains. The NutRich+ as well as LowClim+ diets also aligned well with the EAT-Lancet diet in terms of Dairy foods.

## Discussion

In this study we demonstrated that the combination of cluster analysis with linear optimisation can provide guidance to nutritionally adequate, health-promoting, affordable and climate-friendly diets for different self-selected dietary patterns for the Swedish Population. Our findings show that the three optimised cluster-specific diets differed significantly from the model optimising the average diet of the total population. This novel modelling approach for a climate-friendly and healthy diet may therefore be preferred as it is more consumer oriented. Optimising diets to meet nutritional recommendations and Swedish FBDGs reduced the GHGE by up to 24%. However, this reduction is not sufficient to keep diets within planetary boundaries for climate change. To achieve this goal, the GHGE of the diets would have to be reduced by half compared to baseline. If extrapolating these reductions to the entire adult population in Sweden (~10.4 million), our optimised diets could reduce domestic annual emissions from agricultural food production by roughly 33%, from 6.9 MT [35] to about 4.6 MT. One important strength of our approach is that it leaves a considerable part of the baseline food consumption unchanged while at the same time also reducing cost. The latter might be an additional argument to change diets in times of quickly rising food prices, for example as a result of the 2022 energy crisis.

Similar to what others have found [8, 9, 36–38], the changes seen for all optimised diets were predominantly characterised by shifts from animal products such as red/processed meat, poultry and dairy to plant-based foods such as fruits, vegetables and cereals/grains, albeit to varying degrees depending on the cluster. Particularly, the Classic Baseline pattern had to undergo the most pronounced changes compared to the other two clusters to reach the proposed recommendations and requirements (Fig. 1 and Supplementary Tables 7–10). Besides differing between each other, our findings also show that the three cluster-specific diets (Classic+, NutRich+ and LowClim+) would imply overall dietary shifts that differ from those demanded by the TotPop+ model (Fig. 2). Our results thus indicate that a clustering-optimisation strategy is likely to better capture the dietary heterogeneity that may exist within a delimited context [39]. It is possible that individuals advised to follow a diet that is based on their own specific cluster is more acceptable and thus realistic than a diet optimised on the basis of the national average diet. A similar approach to capture dietary heterogeneity has been applied in the Netherlands [40] where linear programming was used to develop sustainable FBDGs for groups of individuals who consumed meat or not. As the cluster-based optimisation approach considers group-specific preferences, it may make dietary behavioural change more efficient, e.g. by tailoring recommendations/advice to different segments in the population. Naturally, these tailored recommendations should include EER values that may deviate from those calculated for the single clusters. Whether these findings could increase the level of acceptance for climate-friendly diets tailored to different clusters/subgroups in the population remains to be investigated.

The nutritious and health-promoting diets in models TotPop, Classic, NutRich and LowClim were up to 24% lower in GHGE compared to baseline diets. The reduced climate impact from achieving nutritional and health goals aligns with findings from previous research [10, 12, 41, 42]. Yet, our study also shows that switching to a diet meeting only DRVs and the current Swedish FBDGs is not sufficient to keep the climate impact of Swedish diets below the IPCC-suggested CO_2_eq threshold. Such diets were only achievable if the defined GHGE constraint was added to the models (TotPop+, Classic+, NutRich+, LowClim+). As a result, the cost decreased while our proxy for cultural acceptability (the ARD) changed only marginally compared to that observed in the models without a CO_2_eq constraint. In fact, only 5–12% of the foods were changed (either increased/reduced/removed) in the CO_2_eq-constrained diets compared to the baseline diet, indicating that acceptance among consumers within each dietary cluster could be high.

In contrast to other studies from Brazil [43], the US [44], Denmark [29] and Ghana [45] where diets were optimised only to meet nutritional recommendations and FBDGs, the cost of our climate-optimised diets dropped below that of the baseline diet, contradicting assumptions that a healthy, climate-friendly diet is more costly than prevailing food patterns [46] and confirming previous modelling studies indicating lower cost of sustainable nutrition in high-income countries [47].

Our findings reveal that the optimised diets did not align very well with the EAT-Lancet Commission’s dietary recommendation on a sustainable diet. These discrepancies may have several explanations. Firstly, our LP-modelling approach addresses aspects such as a nutrient adequacy (by ensuring the fulfilment of 27 DRVs and the Swedish FBDGs), a shortcoming of the EAT-Lancet diet that already previously has been addressed [48]. Secondly, we implemented dimensions of cultural acceptability (by minimising the TRD and constraining the RD of individual food items) as well as affordability. These aspects are not reported to have been addressed during the design process of the EAT-Lancet diet. Secondly, the food categorisation in the Riksmaten survey includes mixed dishes (wherein e.g. added fats can be “hidden”) whereas the EAT-Lancet diet is composed of “basic” food groups. Hence, the food groups used in Riksmaten are not fully comparable with the EAT-Lancet reference diet’s food groups. Thirdly, in contrast to the optimised diets at hand, the EAT-Lancet diet was developed aiming at health promotion and evaluated against other environmental factors besides GHGE such as water footprint, land use change, and biodiversity. Lastly, the EAT-Lancet diet was developed as a global reference diet and was thus not tailored to a specific national or cultural context. In fact, the authors behind this diet call for cultural and regional adaptations of the dietary recommendations [1]. Hence, the modelling strategy suggested here may be seen as a novel and complementary approach to achieve a cultural tailoring of the EAT-Lancet diet to several distinct subgroups of dietary patterns within a population.

This study assessed the environmental impact of the Swedish diets only on the basis of GHGE, other relevant characteristics of environmental sustainability in the context of diets such as eco-toxicity, land use change, water use, eutrophication, acidification, animal welfare and biodiversity loss were not included due to lack of detailed data for Sweden. Not including these aspects is a limitation since different foods vary in their environmental impacts [49]; animal products tend to be the most GHGE-intense while staple crops (for human consumption), fruits and vegetables, generally are the main contributors to freshwater use per kg of food. However, a drop in GHGE of diets has been observed to be accompanied by substantial reductions in land use and water footprint [50]. Although this study used only the GHGE as an active environmental constraint, it can be assumed that the associated land use and water footprint of the optimised diets are considerably smaller compared to the observed diet.

Our LP modelling did not include foods that were not already present in the baseline diets. There are various new, climate-friendly meat/dairy replacements emerging on the market; many of them fortified with nutrients such as vitamins B12, D and calcium [51, 52]. These are nutrients that tend to be insufficient in plant-based diets. Allowing for these foods to be chosen by the LP-algorithm could be an alternative path to providing climate- and nutrient efficient foods with sensory traits similar to those of animal products. Future optimisations could therefore explore the effects of also including such foods in the modelling as a way to deliver nutritious, climate-friendly and acceptable diet solutions.

This study shows that this novel modelling approach is useful for integrating goals of nutrition, health promotion, climate friendliness and cultural acceptability for different self-selected dietary patterns. Switching to a diet following current nutritional recommendations and Swedish FBDGs is not sufficient to stay below the IPCC CO_2_eq threshold. The fully optimised diets remain within planetary boundaries for climate change while leaving a considerable part of diet unchanged and being lower in cost, suggesting that acceptance among consumers could be high. This is based on the assumption that similarity to existing diets is a predictor of cultural acceptability. The changes seen for all diets were predominantly characterised by shifts from animal products to plant-based foods. However, the shifts required to meet nutrient, FBDG and CO_2_eq constraints varied between the dietary clusters as well as in comparison to the diet optimised for the total population. This suggests that explorative cluster analysis combined with LP is likely to propose dietary shifts that are easier to achieve across a broader range of consumers. The nutritionally adequate, health-promoting and climate-friendly diets in this study did, in various aspects, not match the EAT-Lancet diet. This indicates that there are several approaches through which sustainable diets can be defined, but also that the cultural dietary context plays a bearing role in the optimisation of such diets for specific populations. This study may offer policymakers with insights into how both health promotion and environmental protection may become better connected and thus plausibly also more effective.

### Supplementary information

Supplementary information

## Data Availability

Data can be found within the published article and its supplementary files. Requests for additional materials should be addressed to PEC.

## References

[1] Willett W, Rockström J, Loken B (2019). Food in the Anthropocene: the EAT–Lancet Commission on healthy diets from sustainable food systems. Lancet.

[2] Government Offices of Sweden. Sweden ratifies the climate agreement from Paris. 2016. https://www.regeringen.se/pressmeddelanden/2016/10/sverige-ratificerar-klimatavtalet-fran-paris/

[3] Sarkar A (2022). Addressing consumerism and the planetary health crisis: behavioral economics approach in public policy. Front Energy Res.

[4] Béné C, Fanzo J, Haddad L, Hawkes C, Caron P, Vermeulen S (2020). Five priorities to operationalize the EAT–Lancet Commission report. Nat Food.

[5] Springmann M, Wiebe K, Mason-D’Croz D, Sulser TB, Rayner M, Scarborough P (2018). Health and nutritional aspects of sustainable diet strategies and their association with environmental impacts: a global modelling analysis with country-level detail. Lancet Planet Health.

[6] Gazan R, Brouzes CMC, Vieux F, Maillot M, Lluch A, Darmon N (2018). Mathematical optimization to explore tomorrow’s sustainable diets: a narrative review. Adv Nutr.

[7] Mertens E, Van’t Veer P, Hiddink GJ, Steijns JM, Kuijsten A (2017). Operationalising the health aspects of sustainable diets: a review. Public Health Nutr.

[8] Perignon M, Masset G, Ferrari G, Barré T, Vieux F, Maillot M (2016). How low can dietary greenhouse gas emissions be reduced without impairing nutritional adequacy, affordability and acceptability of the diet? A modelling study to guide sustainable food choices. Public Health Nutr.

[9] Milner J, Green R, Dangour AD, Haines A, Chalabi Z, Spadaro J (2015). Health effects of adopting low greenhouse gas emission diets in the UK. BMJ Open.

[10] Eustachio Colombo P, Patterson E, Elinder LS, Lindroos AK, Sonesson U, Darmon N (2019). Optimizing school food supply: integrating environmental, health, economic, and cultural dimensions of diet sustainability with linear programming. Int J Environ Res Public Health.

[11] Darmon N, Ferguson EL, Briend A (2002). A cost constraint alone has adverse effects on food selection and nutrient density: an analysis of human diets by linear programming. J Nutr.

[12] Horgan GW, Perrin A, Whybrow S, Macdiarmid JI (2016). Achieving dietary recommendations and reducing greenhouse gas emissions: modelling diets to minimise the change from current intakes. Int J Behav Nutr Phys Act.

[13] Maillot M, Vieux F, Amiot MJ, Darmon N (2010). Individual diet modeling translates nutrient recommendations into realistic and individual-specific food choices. Am J Clin Nutr.

[14] Lluch A, Maillot M, Gazan R, Vieux F, Delaere F, Vaudaine S (2017). Individual diet modeling shows how to balance the diet of french adults with or without excessive free sugar intakes. Nutrients.

[15] Cocking C, Walton J, Kehoe L, Cashman KD, Flynn A (2020). The role of meat in the European diet: current state of knowledge on dietary recommendations, intakes and contribution to energy and nutrient intakes and status. Nutr Res Rev.

[16] Gibbons H, Carr E, McNulty BA, Nugent AP, Walton J, Flynn A (2017). Metabolomic-based identification of clusters that reflect dietary patterns. Mol Nutr Food Res.

[17] World Wildlife Fund. One Planet Plate 2019 – kriterier och bakgrund (One Planet Plate 2019 – criteria and background). 2019. https://wwwwwfse.cdn.triggerfish.cloud/uploads/2019/04/kriterier-fr-one-planet-plate-rev-2019.pdf. Accessed 30 Jan 2020.

[18] Amcoff E, Sverige, L. Riksmaten - vuxna 2010-11 Livsmedels- och näringsintag bland vuxna i Sverige. Uppsala: Livsmedelsverket; 2012.

[19] Livsmedelsverket. https://www.livsmedelsverket.se/om-oss/psidata/apimatvanor. Accessed 10 Apr 2020.

[20] Florén B, Amani P, Davis J (2017). Climate database facilitating climate smart meal planning for the public sector in Sweden. Int J Food Syst Dyn.

[21] International Organization for Standardization. ISO 14040:2006 - Environmental management -- Life cycle assessment -- Principles and framework. https://www.iso.org/standard/37456.html. Accessed 9 Oct 2017.

[22] International Organization for Standardization. ISO 14044:2006 - Environmental management -- Life cycle assessment -- Requirements and guidelines. https://www.iso.org/standard/38498.html. Accessed 9 Oct 2017.

[23] Parry ML, Intergovernmental Panel on Climate Change (eds). Climate change 2007: impacts, adaptation and vulnerability: contribution of Working Group II to the fourth assessment report of the Intergovernmental Panel on Climate Change. Cambridge: Cambridge University Press; 2007.

[24] Matpriskollen (Food price check). https://matpriskollen.se/. Accessed 27 Jan 2020.

[25] Brock G, Pihur V, Datta S, Datta S (2008). clValid: an R package for cluster validation. J Stat Softw.

[26] Charrad M, Ghazzali N, Boiteau V (2014). NbClust: an R package for determining the relevant number of clusters in a data set. J Stat Softw.

[27] R Core Team. R: a language and environment for statistical computing; R Foundation for Statistical Computing, Vienna, Austria; 2021.

[28] Moraeus L, Lindroos AK, Warensjö Lemming E, Mattisson I (2020). Diet diversity score and healthy eating index in relation to diet quality and socio-demographic factors: results from a cross-sectional national dietary survey of Swedish adolescents. Public Health Nutr.

[29] Parlesak A, Tetens I, Dejgard Jensen J, Smed S, Gabrijelcic Blenkus M, Rayner M (2016). Use of linear programming to develop cost-minimized nutritionally adequate health promoting food baskets. PLoS ONE.

[30] Dantzig GB (1951). Maximization of a linear function of variables subject to linear inequality. In: Koopmans TC editors. Activity Analysis of Production and Allocation. New York: Wiley; 1947. p. 339–47.

[31] Nocedal J, Wright SJ. Numerical optimization. New York: Springer; 2006.

[32] Mason AJ. OpenSolver - an open source add-in to solve linear and integer progammes in Excel. In: Klatte D, Lüthi H-J, Schmedders K, editors. Operations Research Proceedings 2011. Berlin: Springer; 2012. p. 401–6.

[33] Nordisk Ministerråd. Nordic nutrition recommendations 2012. 5th ed. Copenhagen: Nordic Council of Ministers; 2014.

[34] The Swedish dietary guidelines: find your way to eat greener, not too much and be active. The Swedish Food Agency. Uppsala; 2017.

[35] Territorial emissions and uptake of greenhouse gases. https://www.naturvardsverket.se/data-och-statistik/klimat/vaxthusgaser-territoriella-utslapp-och-upptag. Accessed 15 Mar 2022.

[36] Macdiarmid JI, Kyle J, Horgan GW, Loe J, Fyfe C, Johnstone A (2012). Sustainable diets for the future: can we contribute to reducing greenhouse gas emissions by eating a healthy diet?. Am J Clin Nutr.

[37] Reynolds CJ, Horgan GW, Whybrow S, Macdiarmid JI (2019). Healthy and sustainable diets that meet greenhouse gas emission reduction targets and are affordable for different income groups in the UK. Public Health Nutr.

[38] Broekema R, Tyszler M, van ‘t Veer P, Kok FJ, Martin A, Lluch A, Blonk HTJ (2020). Future-proof and sustainable healthy diets based on current eating patterns in the Netherlands. Am J Clin Nutr.

[39] Vieux F, Perignon M, Gazan R, Darmon N (2018). Dietary changes needed to improve diet sustainability: are they similar across Europe?. Eur J Clin Nutr.

[40] Brink E, van Rossum C, Postma-Smeets A, Stafleu A, Wolvers D, van Dooren C (2019). Development of healthy and sustainable food-based dietary guidelines for the Netherlands. Public Health Nutr.

[41] Green R, Milner J, Dangour AD, Haines A, Chalabi Z, Markandya A (2015). The potential to reduce greenhouse gas emissions in the UK through healthy and realistic dietary change. Clim Change.

[42] Eustachio Colombo P, Elinder LS, Lindroos AK, Parlesak A (2021). Designing nutritionally adequate and climate-friendly diets for omnivorous, pescatarian, vegetarian and vegan adolescents in sweden using linear optimization. Nutrients.

[43] Verly-Jr E, de Carvalho AM, Marchioni DML, Darmon N (2021). The cost of eating more sustainable diets: A nutritional and environmental diet optimisation study. Glob Public Health.

[44] Maillot M, Drewnowski A (2011). Energy allowances for solid fats and added sugars in nutritionally adequate U.S. diets estimated at 17–33% by a linear programming model. J Nutr.

[45] Nykanen E-PA, Dunning HE, Aryeetey RNO, Robertson A, Parlesak A (2018). Nutritionally optimized, culturally acceptable, cost-minimized diets for low income ghanaian families using linear programming. Nutrients.

[46] Barosh L, Friel S, Engelhardt K, Chan L (2014). The cost of a healthy and sustainable diet – who can afford it?. Aust N Z J Public Health.

[47] Springmann M, Clark MA, Rayner M, Scarborough P, Webb P (2021). The global and regional costs of healthy and sustainable dietary patterns: a modelling study. Lancet Planet Health.

[48] Beal T, Ortenzi F, Fanzo J (2023). Estimated micronutrient shortfalls of the EAT–Lancet planetary health diet. Lancet Planet Health.

[49] Springmann M, Clark M, Mason-D’Croz D (2018). Options for keeping the food system within environmental limits. Nature.

[50] Aleksandrowicz L, Green R, Joy EJM, Smith P, Haines A (2016). The impacts of dietary change on greenhouse gas emissions, land use, water use, and health: a systematic review. PLoS ONE.

[51] Franco D, Martins AJ, López-Pedrouso M, Purriños L, Cerqueira MA, Vicente AA (2019). Strategy towards replacing pork backfat with a linseed oleogel in Frankfurter sausages and its evaluation on physicochemical, nutritional, and sensory characteristics. Foods.

[52] Heck RT, Fagundes MB, Cichoski AJ, de Menezes CR, Barin JS, Lorenzo JM (2019). Volatile compounds and sensory profile of burgers with 50% fat replacement by microparticles of chia oil enriched with rosemary. Meat Sci.

[53] Lag om etikprövning av forskning som avser människor (SFS 2003:460) [Law on ethical review of research concerning humans (SFS 2003:460)]. Stockholm: Utbildningsdepartementet; 2003.

