# Supplementary material for: Developing a novel optimisation approach for keeping heterogeneous diets healthy and within planetary boundaries for climate change
Source: Eur J Clin Nutr. 2023 Nov 21;78(3):193–201. doi: 10.1038/s41430-023-01368-7 (PMC10927557; doi:10.1038/s41430-023-01368-7)
Supplement: Supplementary file 1 — Supplementary information [file 41430_2023_1368_MOESM1_ESM.pdf]

Supplementary Information

Developing a novel optimisation approach for keeping heterogeneous diets healthy and within planetary boundaries for climate change

Table of contents

1 Materials and Methods 3

Supplementary Figure 1. Optimal number of clusters when combining Canberra distances with Ward's method. 3

Supplementary Table 1. Baseline intake and ratio between the baseline intake and the recommended intake of different dietary components. 3

Supplementary Table 2. Dietary Reference Values (DRVs) in relation to the baseline average diet of the total study sample (n=1,797) as well of the three clusters, respectively. 4

Dietary data 5

Climate footprints 5

Cost of foods 5

Optimisation 5

1 Results 7

Supplementary Figure 2. Cluster dendrogram showing the hierarchical relationships between the reported intakes of subjects (study participants in Riksmaten Adults, n=1,797) included in the cluster analysis. The distance of split, i.e. height, is shown on the y-axis of the dendrogram. 7

Supplementary Table 3. Median energy-adjusted daily intake of all food groups included in the cluster analysis, median daily CO_2eq, mean healthy eating index score/category, and demographic characteristics for the three clusters. For absolute intake values, see Supplementary Tables 2 and 3. 8

Supplementary Table 4. Daily dietary intake of different food groups, daily dietary CO_2eq, yearly income, age, and sex distribution of the three clusters. 9

Supplementary Table 5. Dietary Reference Values (DRVs) in relation to the optimised diet of the total study sample (n=1,797) as well of the three clusters, respectively, when minimising the total average deviation from baseline intakes as well as applying Dietary Reference Values, the Swedish Food Based Dietary Guidelines and carbon emission limits as constraints. 10

Supplementary Table 6. Absolute quantities of food groups at baseline and after optimising for a nutritionally adequate diet following the Swedish Food Based Dietary Guidelines and generating a maximum of 1571 grams of CO_2eq per day for the four optimised diet-clusters. .. 11

Supplementary Table 7. Quantities of food groups for a nutritionally adequate diet following the Swedish Food Based Dietary Guidelines and generating a maximum of 1571 grams of CO_2eq per day, based on the "TotalPop+"-model. 12

Supplementary Table 8. Quantities of food groups for a nutritionally adequate diet following the Swedish Food Based Dietary Guidelines and generating a maximum of 1571 grams of CO_2eq per day, based on the "Classic+"-model. 12

Supplementary Table 9. Quantities of food groups for a nutritionally adequate diet following the Swedish Food Based Dietary Guidelines and generating a maximum of 1571 grams of CO_2eq per day, based on the "NutRich+"-model. 13

Supplementary Table 10. Quantities of food groups for a nutritionally adequate diet following the Swedish Food Based Dietary Guidelines and generating a maximum of 1571 grams of CO_2eq per day, based on the "LowClim+"-model. 13

References 14

1 Materials and Methods

Supplementary Figure 1. Optimal number of clusters when combining Canberra distances with Ward's method.

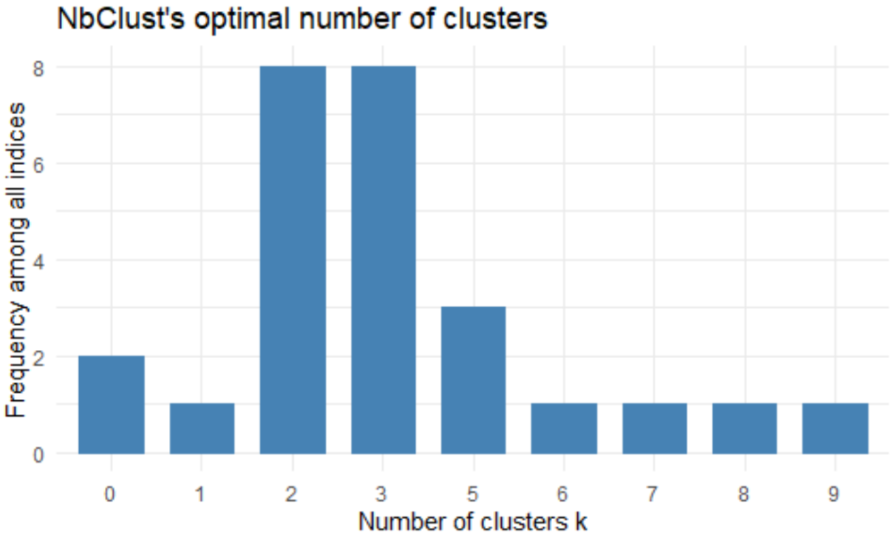

Supplementary Table 1. Baseline intake and ratio between the baseline intake and the recommended intake of different dietary components.

Indicator[a]		Baseline intake			Ratio/score		
		C1	C2	C3	C1	C2	C3
Fruit and vegetables (g/d)[b]	≥500	160	292	302	0.3	0.6	0.6
Fibre (daily E%)	≥2.5	2.0	2.6	2.8	0.8	1.0	1.0
Whole grains (g/10MJ&day)	≥75	33	58	70	0.4	0.8	0.9
Seafood (g/d)[c]	≥45	33	54	52	0.7	1.0	1.0
PUFA (daily (E%)	≥8	6	6	6	0.8	0.8	0.8
MUFA (daily E%)	≥15	13	13	13	0.9	0.9	0.9
SFA daily (daily E%)	≤10	14	13	13	0.6	0.7	0.7
Red/processed meat (g/d)[d]	≤71	115	101	75	1.0	1.0	1.0
Added sugar (daily E%)	≤10	8	8	8	1.0	1.0	1.0
Sum					**7.5**	**6.5**	**7.7**

[a]Based on previous work by Moraeus et al. (1).
[b]Excluding potatoes.
[c]Translates to 2-3 portions á 125 g/week.
[d]Translates to 500 g/week.
C1 = Cluster 1; C2 = Cluster 2; C3 = Cluster 3.

Supplementary Table 2. Dietary Reference Values (DRVs) in relation to the baseline average diet of the total study sample (n=1,797) as well of the three clusters, respectively.

	DRV-constraints[a]		TotPop Baseline		Classic Baseline		NutRich Baseline		LowClim Baseline	
DRV-constraints[a]			% of lower or upper limit of DRV							
	Lower limit	Upper limit	Lower limit	Upper limit	Lower limit	Upper limit	Lower limit	Upper limit	Lower limit	
Energy[b]	Baseline[b]	Baseline[b]	100	100	100	100	100	100	100	100
Protein (E%)	10	20	163	82	156	78	175	88	161	81
Total fat (E%)	25	40	141	88	143	90	138	86	140	88
SFA (E%)	na	10	na	135	na	141	na	128	na	134
PUFA (E%)	5	10	115	58	113	56	116	58	117	59
MUFA (E%)	10	20	132	66	134	67	130	65	130	65
Omega 3 (E%)	1	na	121	na	114	na	125	na	128	na
Carbohydrates (E%)	45	60	95	71	95	71	93	70	97	73
Fibre (g)	25	na	80	na	70	na	82	na	91	na
Added sugar[c]	na	na	na	71	na	82	na	56	na	70
Alcohol (g)	na	15	na	66	na	79	na	67	na	46
Sodium (mg)	na	2400	na	130	na	133	na	130	na	125
Salt (g)	na	6	na	130	na	134	na	130	na	125
Potassium (mg)	1600	na	194	na	187	na	200	na	199	na
Calcium (mg)	500	2500	170	34	173	35	163	33	173	35
Magnesium (mg)	315	2500	104	na	98	na	106	na	109	na
Iron (mg)	11.3	60	92	17	89	17	94	18	94	18
Zinc (mg)	5.5	25	195	43	199	44	196	43	191	42
Selenium (µg)	30	300	152	15	142	14	164	16	154	15
Iodine (µg)	100	600	182	30	169	28	199	33	181	30
Phosphorous (mg)	450	3000	305	46	300	45	309	46	308	46
Vitamin A (RE)	550	3000	149	na	140	na	141	na	170	na
Riboflavin (mg)	1.3	na	122	na	122	na	122	na	122	na
Thiamine (mg)	1.1	na	118	na	117	na	119	na	120	na
Vitamin B6 (mg)	1.2	25	171	8	165	8	179	9	170	8
Vitamin B12 (µg)	1.4	na	391	na	367	na	405	na	409	na
Folate (µg)	200	na	129	na	115	na	136	na	142	na
Vitamin C (mg)	55	1000	172	9	145	8	185	10	194	11
Vitamin D (µg)	7.5	100	93	7	82	6	99	7	100	8
Vitamin E (µg)	5.5	300	225	4	214	4	219	4	246	5
Niacine (NE)	13.5	na	148	na	148	na	160	na	137	na

[a]Based on dietary reference values (estimated energy requirements, recommended intake ranges for macronutrients, and recommended intakes for micronutrients) in the Nordic Nutrition Recommendations 2012 (2).
[b]Set to equal the energy content of the baseline diets (1979, 2068, 1898 and 1942 kcal for total population and Clusters 1-3, respectively.
[c]Set to equal the added sugar intake of the baseline diets (35, 42, 27 and 34 grams for total population and Clusters 1-3, respectively.
DRV = Dietary Reference Value.
na = not applied.

Dietary data

Publicly available data about individual food intake from the dietary survey Riksmaten Adults 2010-11were downloaded from the Swedish Food Agency website (3). In this survey, a nationally representative sample of 5000 individuals aged 18-80 across Sweden were invited to participate (4). The total food and drink intakes were self-reported using by filling in a self-administered online food diary for four consecutive days. The participants were able to choose between ~1900 different food items and dishes and several portion sizes (4). Data regarding age, sex, education and income was acquired through population registers. The publicly available dataset (3) encompasses individual-level data on daily intakes of energy and macro and micronutrients. The dataset also includes each of the reported meal, drink and food item consumed by each participant within the entire recording period (34). Around half of the food in the Riksmaten 2010-11 food composition database were specifically developed for the survey and are not part of the public food composition database. The survey food composition database includes 1909 food items, half of these foods were also included in the public food composition database (n=938) and about half were specifically chosen/built for the survey (n=971). These added foods include combined dishes and food products.

Climate footprints

Factors such as the place of production, and production and transportation methods were part of the quantification of the CO_2eq values. As an example, CO_2eq for a tomato was expressed as an average value from both domestically produced and imported tomatoes, from field or greenhouse production, based on their individual share of the total amount of tomatoes consumed in Sweden. The CO_2eq data did not take into consideration the packaging, transportation from stores to households, meal preparation at home or food waste. For composite foods (e.g. rémoulade sauce), a recipe database from the Swedish Food Agency (Email Lindroos AK, 13th February 2020) was used to calculate CO_2eq values by taking into account proportions of each ingredient. As an example, rémoulade sauce consisting of 200g of mayonnaise with 80% vegetable fat, 18g of drained pickled cucumber and 7g of parsley leaves in the proportions of 0.89, 0.08 and 0.03. These ingredients and amounts were linked with their corresponding CO_2eq values (1.60, 0.08 and 0.35 kg CO_2eq/kg of food item) and summed up resulting in a total CO_2eq value of 1.44 kg CO_2eq/kg of rémoulade sauce.

Cost of foods

The webpage “Matpriskollen” (5), which compares the prices of foods among twelve of Sweden’s largest food retailers, was used to estimate the price of each food. For composite dishes (e.g. lasagne or pancakes) we estimated prices from those pre-made dishes sold commercially and whose prices were available through “Matpriskollen”. in the year 2020. An average price was calculated for each food item based on varying available prices for a food item (including low price, conventional and organic varieties).

Optimisation

As the objective function of all LP models, we chose the minimisation of the total relative deviation (TRD) from the baseline diet (3,4). The TRD from baseline was considered a proxy for cultural acceptability of the optimised diets. The TRD is the (total) sum of the absolute (non-negative) values of the relative deviations (RDs) of the weight of a food in the optimised diet from the reported consumption of this food (Equation 1).

$$\mathrm{RD_i} = \frac{\mathrm{M_i} - \mathrm{m_i}}{\mathrm{m_i}} \qquad (1)$$

In Equation 1, i indicates the running index of the food, M its mass in the optimised diet and m the reported consumption of that food. The TRD from all N food items in the model was calculated as the total sum of the absolute values of RDs:

$$\mathrm{TRD} = \sum_{i=1}^{N} \mathrm{abs}(\mathrm{RD_i}) \quad (2)$$

The TRD is not a linear function and can therefore not be part of a linear equation system such as the one characterising LP. Therefore, the non-negative values of RDi: RD1→RDN, with N being the number of foods included into the optimisation, were generated as described and applied previously (6,7). Briefly, the constraints applied to achieve the optimised absolute RD values were set so that the optimised values were greater or equal to both the actual negative and the positive RD value, resulting in the optimised RD value being equal to the positive RD value, irrespectively whether the deviation was negative (reduced in comparison to the reported consumption) or positive (increased). The decision variables were submitted to the following constraints (Formula 3):

$$\mathrm{abs}(\mathrm{RD_i}) \geq (mi - Mi)/mi \text{ and } \mathrm{abs}(\mathrm{RD_i}) \geq -(mi - Mi)/mi \quad (3)$$

Thus, for each standardised difference, its absolute (positive) value was selected because RDi, by definition, has to be greater than or equal to both the relative difference and its negative value.

To be able to avoid unacceptably high amounts of individual food items in the optimised diets, individual foods were constrained so that they could not increase by more than 200% from baseline

The average relative deviation (ARD) from the baseline food consumption was considered as a proxy of similarity between the baseline and the optimised diets and was calculated by dividing the TRD by the total number of food items included in the model (N), as given in Formula 4:

$$\mathrm{ARD} = \mathrm{TRD/N} \quad (4)$$

1 Results

Supplementary Figure 2. Cluster dendrogram showing the hierarchical relationships between the reported intakes of subjects (study participants in Riksmaten Adults, n=1,797) included in the cluster analysis. The distance of split, i.e. height, is shown on the y-axis of the dendrogram.

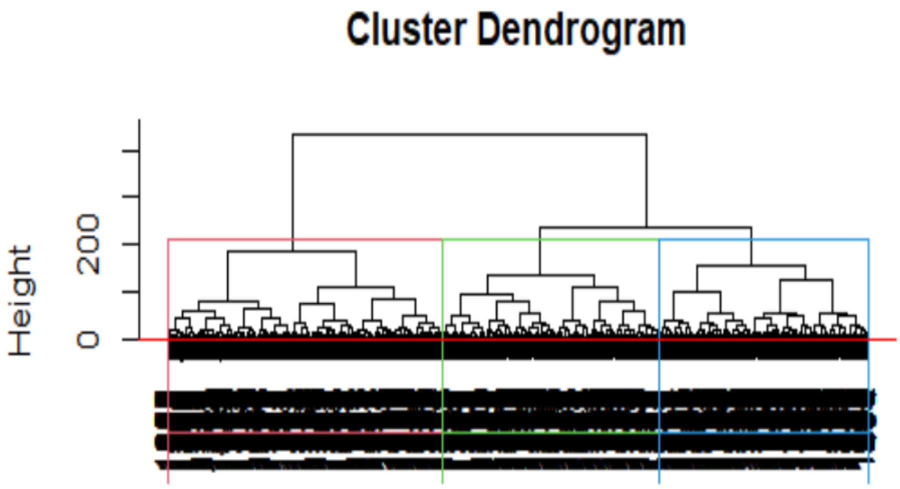

Supplementary Table 3. Median energy-adjusted daily intake of all food groups included in the cluster analysis, median daily CO_2eq, mean healthy eating index score/category, and demographic characteristics for the three clusters. For absolute intake values, see Supplementary Tables 2 and 3.

	Cluster 1	Cluster 2	Cluster 3	Between group sig*
				Cluster number
Red meat$^{(g/MJ)}$	5.7	5.8	3.7	1-3, 2-3
Processed meat$^{(g/MJ)}$	5	4.7	2.8	1-3, 2-3
Poultry$^{(g/MJ)}$	2.4	5.1	1.9	1-2, 1-3, 2-3
Seafood$^{(g/MJ)}$	1.8	6.7	5.5	1-2, 1-3
Dairy$^{(g/MJ)}$	26	33.9	34.9	1-2, 1-3
Vegetables$^{(g/MJ)}$	6.4	15.2	13.9	1-2, 1-3
Fruits and berries$^{(g/MJ)}$	6.1	15.2	18.1	1-2, 1-3
Pulses$^{(g/MJ)}$	0	1.9	2.3	1-2, 1-3
Nuts$^{(g/MJ)}$	0	0.9	0.5	1-2, 1-3
Cereals$^{(g/MJ)}$	16.6	18.7	23.6	1-2, 1-3, 2-3
Rice$^{(g/MJ)}$	3.3	5.5	1.5	1-2, 1-3, 2-3
Potatoes$^{(g/MJ)}$	11.7	10.2	9.9	1 and 2
Sugar and sweets$^{(g/MJ)}$	7.7	5.7	8.2	1-2, 1-3, 2-3
Savoury snacks$^{(g/MJ)}$	0.5	0.3	0.2	1-2, 1-3
CO_2eq$^{(g/MJ)}$	369	372	325	1-3, 2-3
Income$^{(SEK/y)}$	257133	262144	240505	1-3, 2-3
Age$^{(years)}$	44.6	49.4	51.0	1-2, 1-3
HEI$^{(score)}$	6.5	7.7	7.9	na
HEI$^{(category)}$	Medium	High	High	na

*The numbers indicate which clusters that differed significantly (p≤0.05, compared using either the Dunn (1964) Kruskal-Wallis test for multiple comparison or Tukey's honest significance test); blue = highest value; red = lowest value; HEI = Healthy Eating Index; na = not applicable.

Supplementary Table 4. Daily dietary intake of different food groups, daily dietary CO_2eq, yearly income, age, and sex distribution of the three clusters.

	n	mean	sd	min	Q1	median	Q3	max	ZeroCons
Red meat (g/MJ/day)*									
Cluster 1	707	7.2	7.7	0	1.6	5.7	10.3	65.1	20.7
Cluster 2	534	7.2	6.6	0	2.2	5.8	10.6	41.1	18.7
Cluster 3	556	5.5	6.7	0	0	3.7	7.8	68.6	26.8
Vegetables (g/MJ/day)*									
Cluster 1	707	7.9	7.7	0	2.4	6.4	11	60.9	14.4
Cluster 2	534	16.5	11.6	0	9.2	15.2	21.2	87.6	2.6
Cluster 3	556	15.7	12.2	0	7.4	13.9	20.2	104.2	2.3
Fruits and Berries (g/MJ/day)*									
Cluster 1	707	9.5	11.5	0	0	6.1	14.8	114.5	29.3
Cluster 2	534	18.8	15.5	0	7.9	15.2	26.5	84.3	10.1
Cluster 3	556	19.6	14.7	0	8.5	18.1	27.7	112.1	6.5
Dairy (g/MJ/day)*									
Cluster 1	707	32	25.7	0	14.1	26	44.5	156.1	3.1
Cluster 2	534	35.3	21.6	0	20.3	33.9	46	134.2	1.5
Cluster 3	556	36.4	21.9	0	20.5	34.9	48	163.6	1.1
Promeat (g/MJ/day)*									
Cluster 1	707	6.6	6.6	0	1.9	5	9.6	71.2	12.4
Cluster 2	534	5.7	5.2	0	1.6	4.7	8.3	36.2	12
Cluster 3	556	4.1	4.8	0	0.5	2.8	6	47.6	20.9
Pulses (g/MJ/day)*									
Cluster 1	707	0.6	2.7	0	0	0	0	48.7	86.3
Cluster 2	534	1.7	4.2	0	0	0	1.9	48.2	69.1
Cluster 3	556	2	4.2	0	0	0	2.3	42.1	66
Nuts and Seeds (g/MJ/day)*									
Cluster 1	707	0.3	1.1	0	0	0	0	19.2	86.1
Cluster 2	534	0.7	1.4	0	0	0	0.9	14.1	65.9
Cluster 3	556	0.7	1.5	0	0	0	0.5	13.7	67.6
Cereals (g/MJ/day)*									
Cluster 1	707	17.8	8.9	0	12	16.6	21.6	76.2	0.6
Cluster 2	534	21.3	11.6	0	13.5	18.7	26.1	65	0.6
Cluster 3	556	25.1	12.9	0	16.7	23.6	30.6	97.3	0.7
Rice (g/MJ/day)*									
Cluster 1	707	3.3	5.8	0	0	0	5.2	59.2	56.3
Cluster 2	534	5.5	5.6	0	0	4.6	7.7	32.3	30
Cluster 3	556	1.5	3.3	0	0	0	0.5	22.5	74.5
Poatoes (g/MJ/day)*									
Cluster 1	707	14	12.7	0	5.5	11.7	19.6	124.3	10.7
Cluster 2	534	12.1	10.5	0	4.8	10.2	16.2	78.5	12
Cluster 3	556	13.1	12.6	0	4.4	9.9	18.8	125.7	12.8
Poultry (g/MJ/day)*									
Cluster 1	707	2.4	4	0	0	0	3.8	30.6	57.9
Cluster 2	534	5.1	5.2	0	0	4.3	7.5	47.9	28.7
Cluster 3	556	1.9	3.6	0	0	0	2.9	23.9	64.9
Seafood (g/MJ/day)*									
Cluster 1	707	3.8	5.2	0	0	1.8	5.8	32.5	38
Cluster 2	534	7	6.1	0	1	6.7	10.8	37.4	22.8
Cluster 3	556	6.7	6.1	0	2.1	5.5	9.6	44.2	16.2
Sugar and Sweets (g/MJ/day)*									
Cluster 1	707	8.6	7.4	0	3.2	7.7	12.3	67	11.2
Cluster 2	534	6.4	5.7	0	2.7	5.7	8.2	40.4	9.2
Cluster 3	556	9.8	8.7	0	4	8.2	13.5	83.1	7.2
Savoury Snacks (g/MJ/day)*									
Cluster 1	707	0.5	1.3	0	0	0	0.1	14.3	74.1
Cluster 2	534	0.3	0.9	0	0	0	0	7.6	79.2
Cluster 3	556	0.2	0.8	0	0	0	0	7.6	82.9
CO_2eq (g/MJ/day)*									
Cluster 1	707	396	153	172	296	369	462	2004	na
Cluster 2	534	396	120	194	310	372	464	961	na
Cluster 3	556	348	118	106	266	325	409	1116	na
Income (SEK/year)*									
Cluster 1	707	257,438	178,426	0	136,274	257,133	338,933	155,458	4.5
Cluster 2	534	290,748	221,159	0	180,776	262,144	360,001	234,159	2.1
Cluster 3	556	266,555	210,355	0	162,152	240,505	324,098	242,437	1.8
Age* (years)									
Cluster 1	707	44.6	16.5	18	31	44	59	80	na
Cluster 2	534	49.4	15.8	18	37	50	62	80	na
Cluster 3	556	51	16.8	18	38	53	66	80	na
Sex*	%								
Males									
Cluster 1	54								
Cluster 2	38								
Cluster 3	37								

*Significant difference (p≤0.05) between clusters; ZeroCons= percent zero consumers; sd = standard deviation; Q1 = first quartile; Q3 = third quartile.

Supplementary Table 5. Dietary Reference Values (DRVs) in relation to the optimised diet of the total study sample (n=1,797) as well of the three clusters, respectively, when minimising the total average deviation from baseline intakes as well as applying Dietary Reference Values, the Swedish Food Based Dietary Guidelines and carbon emission limits as constraints.

			TotPop+		Classic+		NutRich+		LowClim+	
DRV-constraints[a]			% of lower or upper limit of DRV							
	Lower limit	Upper limit	Lower limit	Upper limit	Lower limit	Upper limit	Lower limit	Upper limit	Lower limit	Upper limit
Energy[b]	na	na	100	100	100	100	100	100	100	100
Protein (E%)	10	20	125	63	123	61	132	66	124	62
Total fat (E%)	25	40	109	68	111	69	108	68	118	74
SFA (E%)	na	10	na	90	na	91	na	89	na	100
PUFA (E%)	5	10	110	55	112	56	108	54	112	56
MUFA (E%)	10	20	104	52	107	53	103	51	114	57
Omega 3 (E%)	1	na	128	na	132	na	127	na	129	na
Carbohydrates (E%)	45	60	120	90	119	89	119	90	118	89
Fibre (g)	25	na	123	na	131	na	123	na	124	na
Added sugar[c]	na	na	na	100	na	100	na	100	na	100
Alcohol (g)	na	15	na	53	na	58	na	44	na	22
Sodium (mg)	na	2400	na	100	na	100	na	100	na	100
Salt (g)	na	6	na	100	na	100	na	100	na	100
Potassium (mg)	1600	na	210	na	222	na	207	na	213	na
Calcium (mg)	500	2500	107	21	114	23	110	22	112	22
Magnesium (mg)	315	2500	111	na	119	na	112	na	113	na
Iron (mg)	11.3	60	100	19	100	19	100	19	100	na
Zinc (mg)	5.5	25	145	32	143	31	147	32	148	33
Selenium (µg)	30	300	118	12	110	11	121	12	120	12
Iodine (µg)	100	600	145	24	162	27	144	24	144	24
Phosphorous (mg)	450	3000	254	38	269	40	256	38	256	38
Vitamin A (RE)	550	3000	142	na	138	na	123	na	151	na
Riboflavin (mg)	1.3	na	103	na	100	na	104	na	103	na
Thiamine (mg)	1.1	na	118	na	124	na	118	na	116	na
Vitamin B6 (mg)	1.2	25	206	10	200	10	208	10	210	10
Vitamin B12 (µg)	1.4	na	320	na	285	na	340	na	336	na
Folate (µg)	200	na	163	na	190	na	154	na	162	na
Vitamin C (mg)	55	1000	284	16	263	14	280	15	297	16
Vitamin D (µg)	7.5	100	101	8	100	7	100	7	100	7
Vitamin E (µg)	5.5	300	236	4	237	4	222	4	251	5
Niacine (NE)	13.5	na	137	na	138	na	134	na	129	na

[a]Based on dietary reference values (estimated energy requirements, recommended intake ranges for macronutrients, and recommended intakes for micronutrients) in the Nordic Nutrition Recommendations 2012 (2).
[b]Set to equal the energy content of the baseline diets (1979, 2068, 1898 and 1942 kcal for total population and Clusters 1-3, respectively.
[c]Set to equal the added sugar intake of the baseline diets (35, 42, 27 and 34 grams for total population and Clusters 1-3, respectively.
DRV = Dietary Reference Values.
na = not applied.

Supplementary Table 6. Absolute quantities of food groups at baseline and after optimising for a nutritionally adequate diet following the Swedish Food Based Dietary Guidelines and generating a maximum of 1571 grams of CO_2eq per day for the four optimised diet-clusters.

	TotalPop Baseline (g)	TotalPop+ (g)	Classic Baseline (g)	Classic+ (g)	NutRich Baseline (g)	NutRich+ (g)	LowClim Baseline (g)	LowClim+ (g)
Red meat	53	12	59	10	56	13	43	11
Processed meat	46	16	56	11	45	18	33	18
Poultry	24	12	20	8	38	11	15	9
Seafood	45	45	33	45	54	49	52	46
Offal	0.3	0.3	0.2	0.2	0.2	0.2	0.4	0.4
Dairy	280	109	275	113	275	130	292	127
Eggs	21	21	16	16	24	24	24	24
Pasta/rice dishes with dairy/eggs	32	32	41	36	24	24	29	29
Pasta/rice dishes with meat/fish	90	52	111	37	73	49	81	51
Vegetables	113	208	76	198	139	166	136	144
Potatoes	94	193	107	222	81	178	89	205
Pulses	10	10	5	79	12	12	16	16
Fruits and berries	120	281	79	224	141	322	150	341
Nuts and seeds	4.5	4.5	2.6	2.6	5.6	5.6	5.9	5.9
Meat substitutes	1.8	1.8	1.6	1.6	0.4	0.4	3.4	3.4
Dairy substitutes	5.5	5.5	2.1	2.1	3.4	3.4	5.8	5.8
Mixed animal fats	11	11	12	15	10	10	12	12
Vegetable fats and oils	0.6	0.6	0.3	0.3	0.7	0.7	0.7	0.7
Cereals/grains	170	265	150	215	169	261	203	218
Rice	28	8	28	8	43	12	12	12
Savoury snacks	3.3	3.3	4.5	4.5	2.8	2.8	2.2	2.2
Sugar and sweets	71	71	77	77	52	52	82	82
Drinks other than milk	792	389	859	446	770	346	728	313
Other	54	51	58	50	49	47	52	52

Supplementary Table 7. Quantities of food groups for a nutritionally adequate diet following the Swedish Food Based Dietary Guidelines and generating a maximum of 1571 grams of CO_2eq per day, based on the “TotalPop+”-model.

- About 260 g of (whole grain) bread and approximately 160 g of other cereals (rice, pasta, etc.) per day
- At least 1 portion (~80 g) of pulses per week
- Around 200 g potatoes per day
- Around 3.5 portions of fruits and 2.5 portions of vegetables per day
- About 3 eggs per week
- 2.5 portions (~315 g) of fish and other seafood per week
- Around 1.5 portions (~280 g) of meat, meat dishes and poultry per week (preferably pork, poultry, and offal such as liver and blood products rather than beef)
- Not more than 110 g of dairy products per day and about 160 g of dairy substitutes per week
- A handful of nuts & seeds per week (~35 g)

Supplementary Table 8. Quantities of food groups for a nutritionally adequate diet following the Swedish Food Based Dietary Guidelines and generating a maximum of 1571 grams of CO_2eq per day, based on the “Classic+”-model.

- About 220 g of (whole grain) bread and approximately 160 g of other cereals (rice, pasta, etc.) per day
- At least 1 portion (~80 g) of pulses per day
- Around 220 g potatoes per day
- Around 3 portions of fruits and 2.5 portions of vegetables per day
- About 2 eggs per week
- 2.5 portions (~315 g) of fish and other seafood per week
- Around 1 portion (~190 g) of meat, meat dishes and poultry per week (preferably pork, poultry, and offal such as liver and blood products rather than beef)
- Not more than 110 g of dairy products per day and about 60 g of dairy substitutes per week
- Half a handful of nuts & seeds per week (~20 g)

Supplementary Table 9. Quantities of food groups for a nutritionally adequate diet following the Swedish Food Based Dietary Guidelines and generating a maximum of 1571 grams of CO_2eq per day, based on the "NutRich+"-model.

- About 260 g of (whole grain) bread and approximately 160 g of other cereals (rice, pasta, etc.) per day
- At least 1 portion (~80 g) of pulses per week
- Around 180 g potatoes per day
- Around 4 portions of fruits and 2 portions of vegetables per day
- About 4 eggs per week
- 2.5 portions (~315 g) of fish and other seafood per week
- Around 1.5 portions (~280 g) of meat, meat dishes and poultry per week (preferably pork, poultry, and offal such as liver and blood products rather than beef)
- Not more than 130 g of dairy products per day and about 100 g of dairy substitutes per week
- A handful of nuts & seeds per week (~35 g)

Supplementary Table 10. Quantities of food groups for a nutritionally adequate diet following the Swedish Food Based Dietary Guidelines and generating a maximum of 1571 grams of CO_2eq per day, based on the "LowClim+"-model.

- About 220 g of (whole grain) bread and approximately 160 g of other cereals (rice, pasta, etc.) per day
- At least 1.5 portions (~120 g) of pulses per week
- Around 200 g potatoes per day
- Around 4.5 portions of fruits and 2 portions of vegetables per day
- About 4 eggs per week
- 2.5 portions (~315 g) of fish and other seafood per week
- Around 1.5 portions (~280) of meat, meat dishes and poultry per week (preferably pork, poultry, and offal such as liver and blood products rather than beef)
- Not more than 130 g of dairy products per day and about 170 g of dairy substitutes per week
- A handful of nuts & seeds per week (~30 g)

References

1. Moraeus L, Lindroos AK, Warensjö Lemming E, Mattisson I. Diet diversity score and healthy eating index in relation to diet quality and socio-demographic factors: results from a cross-sectional national dietary survey of Swedish adolescents. Public Health Nutr 2020;23:1754–65.

2. Nordic nutrition recommendations 2012. 5th ed. Copenhagen: Nordic Council Of Ministers; 2014.

3. Livsmedelsverket [Internet]. [cited 2020 Apr 10]. Available from: https://www.livsmedelsverket.se/om-oss/psidata/apimatvanor

4. Amcoff E, Sverige, Livsmedelsverket. Riksmaten - vuxna 2010-11 Livsmedels- och näringsintag bland vuxna i Sverige [Internet]. Uppsala: Livsmedelsverket; 2012 [cited 2020 Jan 22]. Available from: http://www.slv.se/upload/dokument/rapporter/mat_naring/2012/riksmaten_2010_2011.pdf

5. Matpriskollen (Food price check) [Internet]. [cited 2020 Jan 27]. Available from: https://matpriskollen.se/

6. Darmon N, Ferguson EL, Briend A. A Cost Constraint Alone Has Adverse Effects on Food Selection and Nutrient Density: An Analysis of Human Diets by Linear Programming. J Nutr 2002;132:3764–71.

7. Eustachio Colombo P, Patterson E, Elinder LS, Lindroos AK, Sonesson U, Darmon N, Parlesak A. Optimizing School Food Supply: Integrating Environmental, Health, Economic, and Cultural Dimensions of Diet Sustainability with Linear Programming. Int J Environ Res Public Health 2019;16:3019.
